# Chemical characterization of Nurexan: composition of a multicomponent natural veterinary medicinal product

**DOI:** 10.3389/fvets.2026.1769201

**Published:** 2026-02-20

**Authors:** Stephan Duller, Matthias Bader, Olaf Uhl, Melanie Wergin

**Affiliations:** 1 Elementrial Clinical Research, Graz, Austria; 2 Nuvisan GmbH, Grafing, Germany; 3 Heel GmbH, Baden-Baden, Germany

**Keywords:** flavonoids, natural medicine, Nurexan, phytochemical analysis, stress mitigation, UPLC-HR-MS, veterinary medicine

## Abstract

Nurexan (veterinary formulation; *ad us. vet*.) is a natural medication used to mitigate stress responses in domestic animals. Nurexan contains complex plant extracts from *Passiflora incarnata, Avena sativa*, and *Coffea arabica*, along with the defined chemical compound *Zincum isovalerianicum*. The individual ingredients in Nurexan have each been traditionally used to support stress management, sleep regulation, and mood stabilization through calming, anxiolytic, and neuroactive effects. However, the precise chemical composition of Nurexan and the potential synergistic effects of its ingredients have not yet been fully characterized. This study aimed to identify major chemical constituents in the plant extracts and Nurexan and to quantify selected compounds in the product. Ultra-performance liquid chromatography with high-resolution and tandem mass spectrometry (UPLC-HR-MS, LC-MS/MS) was used to analyze *Coffea arabica, Passiflora incarnata*, and *Avena sativa* extracts as well as Nurexan tablets (*N* = 3 batches). A reversed-phase UPLC gradient with electrospray ionization enabled compound screening, and vitexin and isovitexin were quantified using LC-MS/MS. Chromatographic analysis revealed a complex chemical profile in the plant extracts and Nurexan. Caffeine was detected in *Coffea arabica* and at low levels in Nurexan. Flavonoids including vitexin, isovitexin, orientin, isoorientin, and apigenin, as well as the alkaloid trigonelline, were found in *Passiflora incarnata, Avena sativa*, and Nurexan. Vitexin was present at 1–2 ng per Nurexan tablet, isovitexin at 4–11 ng. Nurexan exhibits a complex composition, including flavonoids with potential pharmacological relevance for stress and sleep regulation. Further studies are needed to elucidate their functional roles.

## 1 Introduction

Nurexan (*ad us. vet*.; referred to hereafter simply as Nurexan) is an established veterinary medicinal product used to mitigate stress responses in horses, pigs, goats, sheep, dogs, and cats. Stress in animals is increasingly recognized as a significant concern due to its detrimental impact on animal welfare. Repetitive exposure to stress poses a serious risk for various mental and physical health issues, including gastritis, gastric ulcerations, immunosuppression, or anxiety disorders. Clinical evidence for Nurexan's efficacy is derived mainly from studies with Neurexan, the human medical equivalent, which has the same formulation and follows an identical manufacturing process. Neurexan's anti-stress effects have been demonstrated in a pivotal randomized controlled trial (RCT) conducted in high-performance Siberian sled dogs undergoing rigorous training for racing. This study showed that Neurexan significantly reduced the exercise-induced increase in cortisol and gastrin (1).

In humans, Neurexan is used as a pharmacological option to mitigate symptoms associated with everyday stress, such as nervous restlessness (2), and related sleep disturbances (3). Numerous studies showed the biological effects of Neurexan as well as its therapeutic efficacy. In a preclinical study, Neurexan mitigated acute stress-induced insomnia in rats exposed to a psychosocial stressor (4). An RCT demonstrated that Neurexan diminished the increase in salivary cortisol and plasma adrenaline in response to the Trier Social Stress Test in healthy participants (5). Another RCT tested Neurexan's effects on mildly to moderately stressed individuals during resting states and while performing attentional, emotional, and stress-related tasks. This study showed that Neurexan reduced neural stress network activation in response to a stress task (6), dampened resting brain activity after stress in individuals with high trait anxiety (7), improved vigilance regulation during recovery from stress (8), and enhanced EEG and heart rate variability-based stress biomarkers in the recovery phase (9). Additional findings include reduced amygdala activation in response to negative emotional stimuli (10), modulation of task-free resting-state functional connectivity, suggesting improved emotion regulation (11), and a reduction in distractibility in an attention modulation task (12). Real-world evidence further supports the effectiveness of Neurexan in reducing stress-related symptoms. Two observational studies demonstrated its benefits in patients with insomnia (3) and in those experiencing nervous restlessness (2). A recent review conceptualized Neurexan's mode of action as mitigating the impact of stress on the negative valence system while improving arousal and cognition, thereby contributing to the restoration of overall system functionality (13).

Nurexan is a multicomponent tablet formulation produced under German pharmaceutical regulations and in full compliance with international Good Manufacturing Practice (GMP) standards (14, 15). Although regulated within the homeopathic framework, the formulation contains low-concentration plant-derived extracts and a defined inorganic compound, resulting in the presence of analytically measurable phytochemical constituents. It comprises extracts of *Passiflora incarnata* (purple passionflower), *Avena sativa* (common oats), and *Coffea arabica* (coffee plant), as well as synthetically derived *Zincum isovalerianicum* (valerianate of zinc). *Passiflora incarnata* and *Avena sativa* have traditionally been used for stress and sleep support (16, 17). Low doses of caffeine from *Coffea arabica* have been reported to improve hedonic tone and reduce anxiety (18, 19). *Zincum isovalerianicum* (Zn(C_5_H_9_O_2_)_2_·2H_2_O) is traditionally used for its calming, antispasmodic, and anxiolytic effects, combining zinc's role in modulating neuronal activity with the anxiety-reducing properties of isovaleric acid, a compound naturally found in valerian root (20–22).

Nurexan's multi-component, multi-target approach aligns with the concept of network pharmacology, which emphasizes the modulation of multiple targets within complex biological systems to address multifactorial pathophysiologies (23). Although clinical data from the corresponding human formulation support its efficacy, its detailed chemical composition remains largely unresolved due to the inherent complexity of plant extracts and the high dilution levels used in the formulation. While the identity and concentration of *Zincum isovalerianicum* in the tablets are well defined by the manufacturing process, the chemical profiles of the three plant extracts–*Passiflora incarnata, Avena sativa*, and *Coffea arabica*–remain insufficiently characterized, particularly with regard to their active constituents and their potential pharmacological relevance.

Recent advances in analytical separation techniques and mass spectrometry (MS) have significantly improved the sensitivity, selectivity, and resolution required for the qualitative and quantitative analysis of complex herbal mixtures (24). In particular, MS has become an indispensable tool for the identification of low-abundance constituents, structural elucidation of unknown compounds, and the detection of chemically diverse metabolites present in multi-component herbal formulations. These capabilities are essential when working with natural extracts, which often contain hundreds of structurally related compounds at varying concentration levels, many of which may contribute to the overall pharmacological activity. Depending on the instrumentation and matrix, modern MS techniques can reliably detect compounds in the low nanogram to high picogram range, making them well suited for profiling minor constituents in complex botanical products.

In line with the concept of plant-based, multicomponent therapies in veterinary medicine, a detailed phytochemical characterization of Nurexan is needed to better understand its composition. The objective of this study was therefore to provide a descriptive and exploratory characterization of the plant-derived chemical composition of Nurexan and the individual plant extracts used in its formulation using UPLC-HR-MS, and to quantify selected flavonoid markers in both the extracts and the finished tablets. This work was designed to focus on chemical profiling and quantification and was not intended to assess biological efficacy or therapeutic effectiveness.

## 2 Materials and methods

### 2.1 Study materials and product description

Nurexan is a veterinary medicinal product in tablet form, manufactured in accordance with German pharmaceutical regulations and international GMP standards. Ingredients of Nurexan are *Passiflora incarnata* (purple passionflower) D2 0.6 mg, *Avena sativa* (common oats) D2 0.6 mg, *Coffea arabica* (coffee plant) D12 0.6 mg, and *Zincum isovalerianicum* (valerianate of zinc) D4 0.6 mg. In addition, carriers incorporated in the tablets are small amounts of lactose monohydrate and magnesium stearate.

Plant extracts from *Coffea arabica* (batch: 666666), *Passiflora incarnata* (batches: 626120 and 627453), and *Avena sativa* (batches: 659681 and 642583) were received from Heel GmbH (Baden-Baden, Germany). The botanical identity and quality of all plant materials were verified in accordance with the monographs of the German Homeopathic Pharmacopeia (15), either by the certified tincture manufacturers or by the product manufacturer during mother tincture preparation. Extracts (in ethanol-water mixture) were prepared from the fresh aerial parts of *Passiflora incarnata*, the fresh aerial parts during flowering of *Avena sativa*, and the dried, unroasted seeds of *Coffea arabica*. Three batches of Nurexan tablets (05827, 02620, and 02618) were also received from Heel GmbH (Baden-Baden, Germany). Reference compounds included vitexin, isovitexin, vitexin-2′-O-rhamnoside, orientin, isoorientin, schaftoside, isoschaftoside, isoscoparin, apigenin, trigonelline, caffeine, theobromine, caffeic acid, gallic acid, salicylic acid, cafestol, lutonarin, and gamma-oryzanol. The fourth ingredient of Nurexan, *Zincum isovalerianicum*, was not analyzed in the scope of this study, as its content is precisely defined during the production process and amounts to 0.06 μg per tablet.

### 2.2 Screening of plant extracts and Nurexan

Screening and identification of the main constituents in plant extracts (*Coffea arabica, Passiflora incarnata*, and *Avena sativa*) and Nurexan tablets were performed using ultra-performance liquid chromatography coupled with high-resolution mass spectrometry (UPLC-HR-MS).

The *Coffea arabica* extract was diluted 1 + 9 (V/V) with acetonitrile/methanol/water (1 + 1 + 8, V/V/V). Diluted and undiluted extracts of *Coffea arabica*, as well as extracts of *Passiflora incarnata* and *Avena sativa*, were injected directly into the UPLC-HR-MS system. Nurexan tablets (approximately 0.9 g) were dissolved in 5 mL ethanol and homogenized for 30 s (three cycles) using a Precellys system. The homogenate was centrifuged for 5 min at 13,000 × g, and 2.5 mL of each supernatant was evaporated to dryness under a stream of nitrogen at 37 °C. The residue was redissolved in 250 μL acetonitrile/methanol/water (1 + 1 + 8, V/V/V), sonicated for 10 min, and centrifuged for 5 min at 13,000 × g before injection.

UPLC-HR-MS analysis was conducted using a Waters Acquity UPLC system coupled with a Waters Vion IMS quadrupole time-of-flight (QTOF) mass spectrometer (Waters, Eschborn, Germany) equipped with an electrospray ionization (ESI) interface. Data were processed using Unifi software (version 1.9.4).

Chromatographic separation was performed using a Waters HSS T3 column (1.8 μm, 3.0 × 150 mm) at a column temperature of 45 °C. The autosampler was maintained at 10 °C. The mobile phases consisted of 0.1% formic acid in water (A) and 0.1% formic acid in acetonitrile (B), with a flow rate of 0.7 mL/min. The gradient started at 95% A and 5% B for 5.0 min, then B was increased to 50% at 30.0 min and further to 95% at 35.0 min, where it remained until 40.0 min. At 40.1 min, the initial conditions were restored and maintained until 45.0 min.

The mass spectrometer was operated in both positive and negative ionization modes. The capillary voltage was set to 2.0 kV, the sampling cone to 40 V, and the source offset to 80 V. The source and desolvation temperatures were 120 °C and 600 °C, respectively. The mass range was set to 50–1,300 Da, with collision energies ranging from 10 to 30 V.

UPLC-HR-MS screening analyses were performed as single technical measurements per prepared sample and served as a non-targeted, qualitative approach for compound identification. No lower limit of quantification (LLOQ) was defined.

### 2.3 Determination of vitexin and isovitexin

The quantification of vitexin and isovitexin in Nurexan tablets and plant extracts was performed using LC-MS/MS. Vitexin and isovitexin were selected due to their known high abundance and significant biological activities in both *Passiflora incarnata* and *Avena sativa*, making them suitable marker compounds despite the presence of other bioactive constituents. Stock solutions of vitexin and isovitexin (500 μg/mL) were prepared in dimethylformamide and stored at 4 °C in glass containers. Quercetin-3-D-galactoside was used as the internal standard.

For sample preparation, 1 g of Nurexan tablet material was weighed into a 15 mL Precellys tube and homogenized with 4 mL acetonitrile/water (1 + 9, V/V) at 5,500 rpm for three cycles of 30 s, with 30 s pause times. The mixture was centrifuged at 10,000 × g for 5 min, and the supernatant was transferred into a new Eppendorf tube. A second centrifugation was performed before injection into the LC-MS/MS system. *Passiflora incarnata* extract was diluted 1:100,000, and *Avena sativa* extract was diluted 1:1,000 in acetonitrile/water (1 + 9, V/V) before injection.

Chromatographic separation was performed using a Waters Acquity HSS T3 column (100 × 2.1 mm, 1.8 μm) at 40 °C with a flow rate of 0.8 mL/min. The mobile phases consisted of 0.1% formic acid in water (A) and 0.1% formic acid in acetonitrile (B). The gradient started at 98% A and 2% B for 1.0 min, then B increased to 20% at 10.0 min and to 95% at 12.0 min, which was maintained until 13.0 min. At 13.1 min, the initial conditions were restored and held until 15.0 min.

The AB Sciex Triple Quadrupole MS-Detector API 5500 was used in negative ESI mode, applying multiple reaction monitoring. Mass transitions were set as follows: vitexin, 431.0 → 311.0; isovitexin, 431.0 → 283.0; and internal standard, 463.0 → 270.9. The source temperature was maintained at 650 °C, with nebulizer and turbo gas pressures of 60 and 65 psi, respectively. A calibration curve ranging from 0.05 to 25.0 ng/mL was established using linear regression with 1/*x*^2^ weighting. Data were analyzed using Applied Biosystems Analyst software.

For LC-MS/MS quantification, Nurexan tablet samples were analyzed in three technical replicates per batch, whereas plant extracts were measured as single technical replicates due to their use as qualitative and comparative reference materials. Analytical variability for vitexin and isovitexin in Nurexan tablets was low, with coefficients of variation (CV%) ranging from 0.62 to 2.45 for vitexin and from 0.95 to 3.55 for isovitexin across batches.

The LLOQ for both vitexin and isovitexin was defined as the lowest calibration standard and amounted to 0.05 ng/mL.

Due to the exploratory nature of the study and the limited number of batches analyzed, no inferential statistical analyses were intended or performed; results are presented descriptively.

## 3 Results

### 3.1 Screening of plant extracts and Nurexan

The plant extracts of *Coffea arabica, Passiflora incarnata*, and *Avena sativa*, as well as Nurexan tablets, were analyzed using UPLC-HR-MS to identify and confirm the presence of characteristic phytochemicals. As summarized in Table 1, the screening detected caffeine, theobromine, trigonelline, and caffeic acid consistent with *Coffea arabica*; a panel of flavone C-glycosides in *Passiflora incarnata* and *Avena sativa*–including vitexin, isovitexin, vitexin-2′-O-rhamnoside, orientin, isoorientin, schaftoside, isoschaftoside, isoscoparin, and apigenin; and several of these marker compounds (e.g., trigonelline, caffeine, vitexin, isovitexin, orientin, isoorientin, schaftoside, isoschaftoside, isoscoparin, apigenin) in Nurexan. Among the reference compounds investigated, salicylic acid, gallic acid, cafestol, lutonarin, and gamma-oryzanol were not detected in any of the plant extracts or Nurexan tablet batches.

**Table 1:** Occurrence of selected compounds in plant extracts and Nurexan.

**Compound**	***Avena sativa***	***Passiflora incarnata***	***Coffea arabica***	**Nurexan**
**Flavonoids**				
Vitexin	+	+	–	+
Isovitexin	+	+	–	+
Vitexin-2′-O-rhamnoside	+	+	–	–
Orientin	–	+	–	+
Isoorientin	–	+	–	+
Schaftoside	–	+	–	+
Isoschaftoside	+	+	–	+
Isoscoparin	+	+	–	+
Apigenin	+	+	–	+
**Alkaloids**				
Caffeine	–	–	+	+
Theobromine	–	–	+	–
Trigonelline	+	+	+	+
**Phenolic acids**				
Caffeic acid	–	–	+	–

Additional reference compounds salicylic acid, gallic acid, cafestol, lutonarin, and gamma-oryzanol were included in the screening but were not detected in any of the plant extracts or Nurexan tablet batches. +: detected, –: not detected.

**Figure 1:**
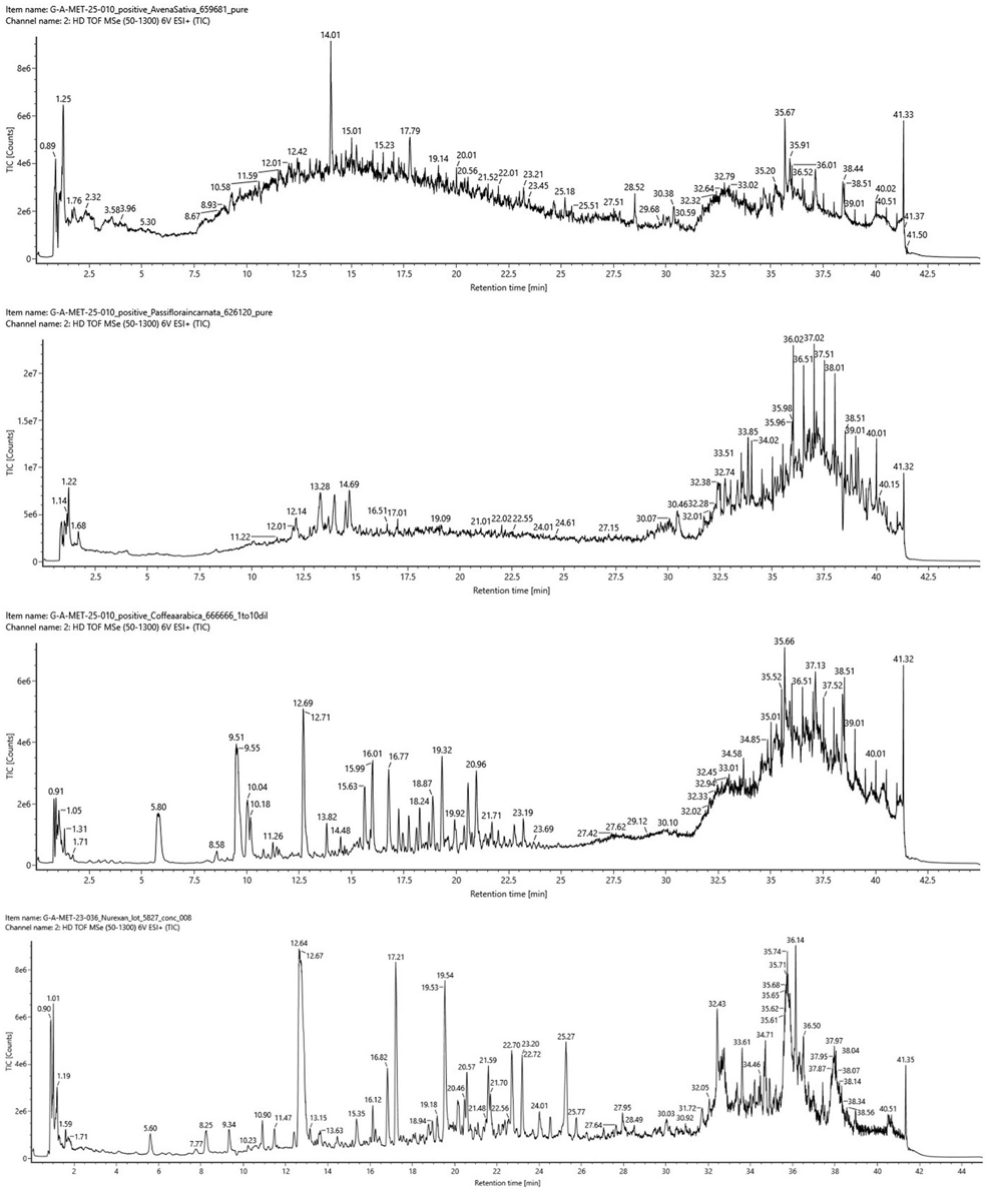
Total ion chromatograms (TICs) obtained by UPLC-HR-MS in electrospray ionization (ESI) positive mode for *Avena sativa, Passiflora incarnata*, and *Coffea arabica* extracts and Nurexan tablets (batch 05827; from top to bottom). The chromatograms illustrate the overall chemical complexity of the individual plant extracts and the presence of corresponding signals in the final Nurexan formulation.

Total ion chromatograms (TICs) in positive and negative electrospray ionization (ESI) modes revealed distinct chemical profiles for each extract (Figure 1). The TICs showed multiple peaks corresponding to diverse metabolites, with noticeable differences in peak intensities and retention times among the plant extracts. The TICs for Nurexan tablets exhibited signals corresponding to compounds detected in the plant extracts, confirming their presence in the final formulation.

**Figure 2:**
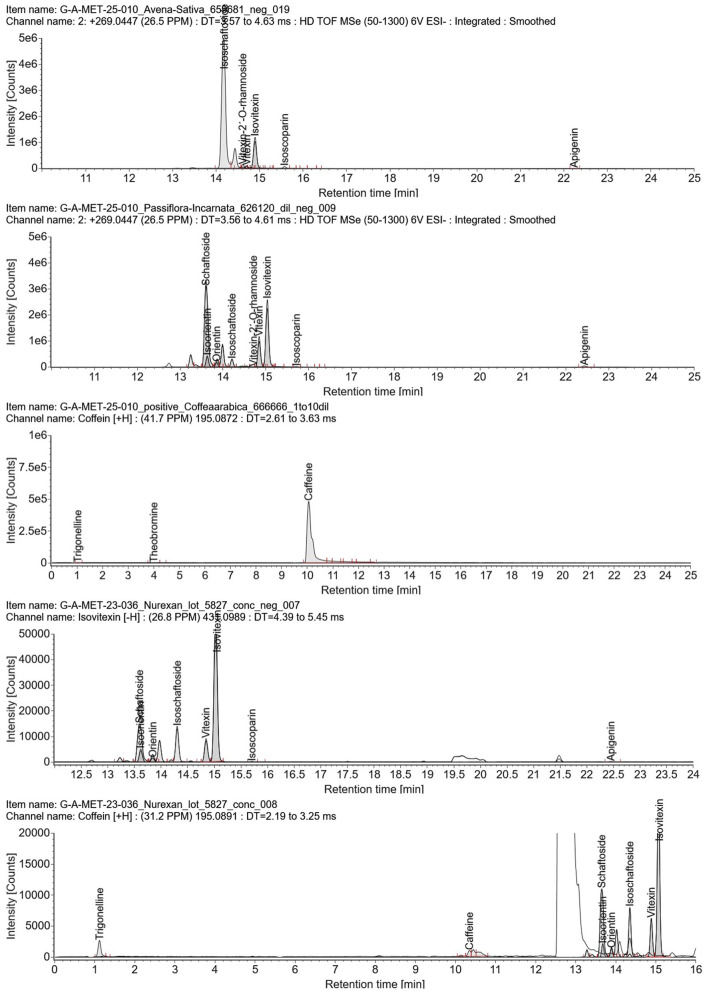
Extracted ion chromatograms (EICs) obtained by UPLC-HR-MS for representative marker compounds in *Avena sativa* and *Passiflora incarnata* extracts (ESI negative mode), *Coffea arabica* extract (ESI positive mode), and Nurexan tablets (batch 05827; ESI negative and positive modes). Peak labels indicate the main flavonoid and alkaloid markers in each chromatogram, illustrating the characteristic phytochemical profiles of the plant extracts and their transfer into the final Nurexan formulation.

**Figure 3:**
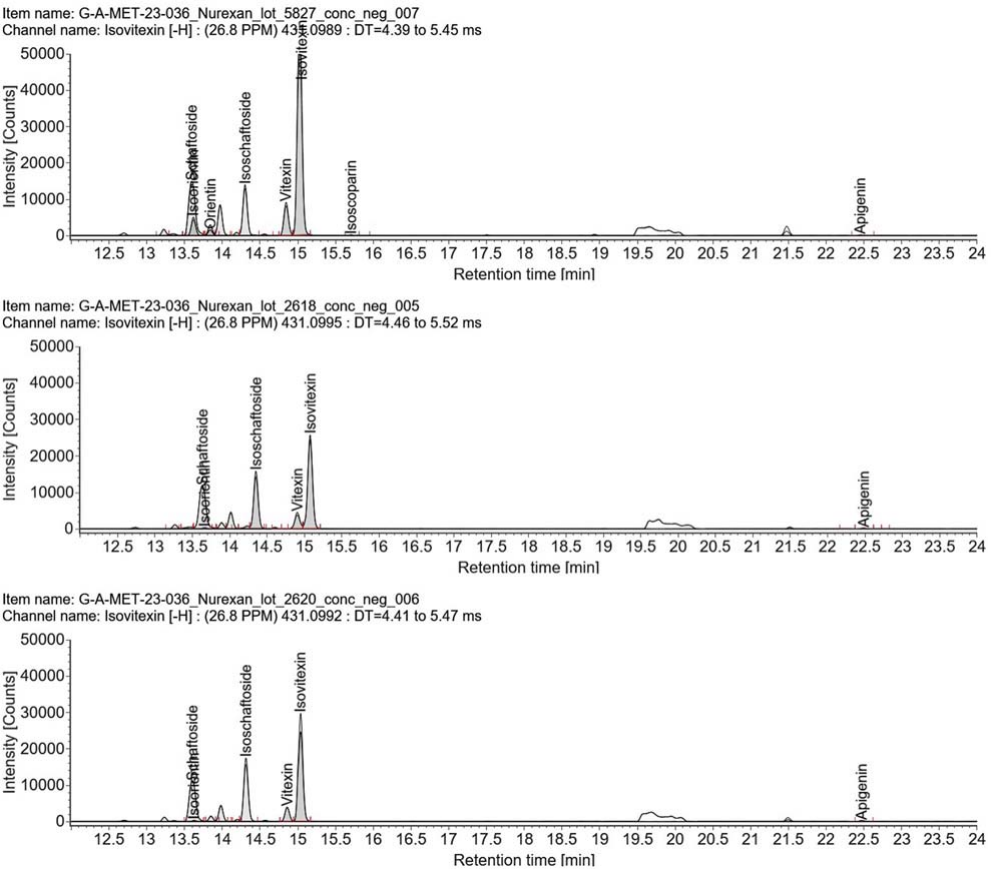
Extracted ion chromatograms (EICs) obtained by UPLC-HR-MS in electrospray ionization (ESI) negative mode for Nurexan tablets from three production batches (from top to bottom: 05827, 02618, 02620). Peaks corresponding to the flavone C-glycosides and related flavonoids schaftoside, isoorientin, orientin, isoschaftoside, vitexin, isovitexin, and isoscoparin, as well as apigenin, are labeled. All three batches display a comparable overall flavonoid pattern. Orientin and isoscoparin are evident only in batch 05827, illustrating batch-dependent variability in selected polyphenols.

Extracted ion chromatograms (EICs) provided further confirmation of the target compounds (Figure 2). Caffeine was detected in *Coffea arabica* and in Nurexan. Vitexin and isovitexin were present in both *Passiflora incarnata* and *Avena sativa*, and were also detected in Nurexan tablets. In addition, other polyphenols characteristic of *Passiflora incarnata* and *Avena sativa*–including orientin, isoorientin, schaftoside, isoschaftoside, isoscoparin, and apigenin–were observed in Nurexan tablets (Table 1). Orientin and isoscoparin were detected in Nurexan batch 05827, whereas these compounds were not found in batches 02618 and 02620 (Figure 3).

### 3.2 Quantification of vitexin and isovitexin

The concentrations of vitexin and isovitexin in Nurexan tablets (three batches) and in plant extracts of *Passiflora incarnata* and *Avena sativa*, two batches each, were determined using LC-MS/MS. As expected, *Passiflora incarnata* showed the highest concentrations of both flavonoids, with vitexin ranging from 79.0 to 90.1 mg/L and isovitexin from 353 to 501 mg/L. *Avena sativa* contained lower amounts overall, with vitexin between 0.48 and 2.58 mg/L and isovitexin between 11.4 and 57.6 mg/L. For *Avena sativa*, there was marked variability between the two batches: batch 642583 contained approximately fivefold higher vitexin and isovitexin levels than batch 659681 (Table 2).

**Table 2:** Concentration of vitexin and isovitexin in plant extracts (mg/L) and Nurexan tablets (ng/tablet).

**Compound**	***Avena sativa***		***Passiflora incarnata***		Nurexan		
	**lot 659681**	**lot 642583**	**lot 626120**	**lot 627453**	**lot 02618**	**lot 02620**	**lot 05827**
Vitexin	0.48	2.58	90.1	79.0	0.95 (0.01)	1.02 (0.02)	2.11 (0.05)
Isovitexin	11.4	57.6	501	353	3.94 (0.04)	4.10 (0.15)	11.5 (0.38)

Plant extracts were measured in single technical replicates; Nurexan tablets were measured in three technical replicates per lot. Results for Nurexan are reported as mean (standard deviation, n = 3).

Analysis of Nurexan tablets confirmed the presence of these flavonoids, indicating their successful transfer from the plant extracts into the final formulation. The concentration of vitexin in the tablets ranged from 0.95 to 2.11 ng/tablet, and isovitexin concentrations varied between 3.94 and 11.5 ng/tablet. Among the tested batches, batch 05827 contained the highest amounts of both flavonoids (2.11 ng/tablet vitexin and 11.5 ng/tablet isovitexin), whereas batches 02618 and 02620 showed lower but similar concentrations (Table 2).

These findings confirm the incorporation of plant-derived flavonoids into the Nurexan formulation. As expected, the concentrations of vitexin and isovitexin in the final product were markedly lower than those in the raw plant extracts, consistent with dilution during the manufacturing process. The overall pattern of higher levels in *Passiflora incarnata* compared to *Avena sativa* was maintained across plant extracts and tablet batches.

## 4 Discussion

The UPLC-HR-MS analysis confirmed the presence of known bioactive compounds in the plant extracts and their successful incorporation into Nurexan tablets. Caffeine, a well-known stimulant, was detected in *Coffea arabica* and at very low levels in Nurexan, consistent with the high dilution factor of the ingredient in the final product. Notably, low concentrations of caffeine derived from *Coffea arabica* have been associated with enhanced hedonic tone and reduced anxiety levels (18, 19). Similarly, vitexin and isovitexin, characteristic flavonoids of *Passiflora incarnata* and *Avena sativa*, were present in both the raw extracts and the final tablet formulation, albeit at lower concentrations, which can be attributed to the dilution effects inherent to the manufacturing process.

Vitexin, a flavonoid found in various medicinal plants, has been widely reported to exhibit anxiolytic and anticonvulsant properties (25, 26). These effects appear to be primarily mediated through modulation of GABAergic neurotransmission. In line with the proposed vitexin effect, previous studies have reported an affinity of Neurexan to the central GABA_A_ receptor (27). In animal models, vitexin demonstrated dose-dependent protection against seizures induced by GABA_A_ receptor antagonists such as picrotoxin and pentylenetetrazol, suggesting a selective action on GABAergic pathways (28). Behavioral studies such as the elevated plus maze and open field test further support its anxiolytic activity, with treated animals showing increased open-arm exploration without sedation. In addition to its GABAergic effects, vitexin has been reported to modulate serotonergic and adrenergic systems, contributing to its antiallodynic and antihyperalgesic effects (29). Together, these mechanisms support vitexin's potential contribution to the overall pharmacological activity of Nurexan, particularly in modulating anxiety, seizure susceptibility, and pain-related symptoms.

In addition to the target compounds, screening revealed the presence of other polyphenols in the plant extracts, notably orientin and related flavonoids. These findings align with literature reports on the phytochemical composition of *Passiflora incarnata* and *Avena sativa*, suggesting that additional secondary metabolites may contribute to the overall pharmacological properties of Nurexan. Orientin, a flavone C-glycoside, has been identified in *Passiflora incarnata* and is known for its antioxidant and neuroprotective activities, potentially enhancing the anxiolytic effects of the formulation (30–32). Apigenin, another flavonoid detected in the extracts, has been shown to bind to central benzodiazepine receptors and to exert anxiolytic activity without sedative or myorelaxant effects (33). It has additionally been linked to modulation of GABAergic and monoaminergic pathways and to improvements in stress- and inflammation-related behavioral alterations (34). Trigonelline, an alkaloid present in all three plant extracts and consistently detected in Nurexan tablets, has likewise been described as exhibiting neuroprotective and neuromodulatory properties, particularly through attenuation of oxidative and inflammatory stress responses (35). These actions suggest that trigonelline may contribute to the formulation's anti-stress profile by supporting neurochemical stability under challenging conditions (35).

The presence of these compounds underscores the complexity of the herbal extracts and the potential synergistic effects contributing to the formulation's overall activity. The batch-dependent detection of some polyphenols such as orientin and isoscoparin in Nurexan is consistent with the natural variability expected for multi-component herbal formulations and may modulate the relative contribution of individual constituents without altering the overall phytochemical fingerprint. Conversely, several additional reference compounds that were included in the analytical panel (salicylic acid, gallic acid, cafestol, lutonarin, and gamma-oryzanol) were not detected in any of the tinctures or tablet batches under the present conditions, indicating that they are unlikely to contribute meaningfully to the pharmacological profile of Nurexan.

Although the concentrations of vitexin and isovitexin in Nurexan are markedly lower than in the corresponding plant extracts, their consistent detectability supports the formulation's botanical origin and is in line with the expected dilution effects of the manufacturing process. The observed lot-to-lot variability falls within the typical range for natural products. Further studies are warranted to assess the potential synergistic interactions among these constituents and to clarify their bioavailability *in vivo*.

In addition to the plant-derived components, the fourth ingredient of Nurexan, *Zincum isovalerianicum*, though not analyzed in the scope of this study due to its known concentration, may also contribute to the overall pharmacological profile of the formulation. Traditionally used for its calming, antispasmodic, and anxiolytic effects, this compound combines the neuromodulatory role of zinc with the sedative and anxiety-reducing properties of isovaleric acid–a constituent of valerian root (20–22). Its potential synergistic action with the plant-based constituents warrants further investigation to fully understand the multimodal activity of Nurexan.

Overall, these results provide a detailed chemical characterization of Nurexan and its plant-based components, offering insight into its formulation and establishing a compositional basis for future investigations into its potential biological relevance.

## Data Availability

The raw data supporting the conclusions of this article will be made available by the authors, without undue reservation.
